# Novel BRICHOS-Related Antimicrobial Peptides from the Marine Worm *Heteromastus filiformis*: Transcriptome Mining, Synthesis, Biological Activities, and Therapeutic Potential

**DOI:** 10.3390/md21120639

**Published:** 2023-12-14

**Authors:** Pavel V. Panteleev, Victoria N. Safronova, Shuting Duan, Alexey S. Komlev, Ilia A. Bolosov, Roman N. Kruglikov, Tatiana I. Kombarova, Olga V. Korobova, Eugenia S. Pereskokova, Alexander I. Borzilov, Igor A. Dyachenko, Olga V. Shamova, Yu Huang, Qiong Shi, Tatiana V. Ovchinnikova

**Affiliations:** 1 M.M. Shemyakin & Yu.A. Ovchinnikov Institute of Bioorganic Chemistry, Russian Academy of Sciences, 117997 Moscow, Russia; p.v.panteleev@gmail.com (P.V.P.); victoria.saf@ibch.ru (V.N.S.); duanshuting@yandex.ru (S.D.); bolosov@ibch.ru (I.A.B.); kruglikov1911@mail.ru (R.N.K.); 2 Shenzhen Key Lab of Marine Genomics, Guangdong Provincial Key Lab of Molecular Breeding in Marine Economic Animals, BGI Academy of Marine Sciences, BGI Marine, Shenzhen 518081, China; huangyu@genomics.cn (Y.H.); shiqiong@genomics.cn (Q.S.); 3 Institute of Experimental Medicine, WCRC “Center for Personalized Medicine”, 197022 St. Petersburg, Russia; komlev1420@yandex.ru (A.S.K.); oshamova@yandex.ru (O.V.S.); 4 State Research Center for Applied Microbiology & Biotechnology (SRCAMB), 142279 Obolensk, Russia; kombarova@obolensk.org (T.I.K.); korobova@obolensk.org (O.V.K.); pereskokova@obolensk.org (E.S.P.); borzilov@obolensk.org (A.I.B.); 5 The Branch of M.M. Shemyakin & Yu.A. Ovchinnikov Institute of Bioorganic Chemistry, Russian Academy of Sciences, 142290 Pushchino, Russia; dyachenko@bibch.ru; 6 Laboratory of Aquatic Genomics, College of Life Sciences and Oceanography, Shenzhen University, Shenzhen 518057, China; 7 Department of Biotechnology, I.M. Sechenov First Moscow State Medical University, 119991 Moscow, Russia

**Keywords:** antimicrobial peptide, Annelida, Polychaeta, *Heteromastus filiformis*, transcriptome mining, BRICHOS domain, recombinant peptide, murine models, antimicrobial resistance, antibiofilm activity

## Abstract

Marine polychaetes represent an extremely rich and underexplored source of novel families of antimicrobial peptides (AMPs). The rapid development of next generation sequencing technologies and modern bioinformatics approaches allows us to apply them for characterization of AMP-derived genes and the identification of encoded immune-related peptides with the aid of genome and transcriptome mining. Here, we describe a universal bioinformatic approach based on the conserved BRICHOS domain as a search query for the identification of novel structurally unique AMP families in annelids. In this paper, we report the discovery of 13 novel BRICHOS-related peptides, ranging from 18 to 91 amino acid residues in length, in the cosmopolitan marine worm *Heteromastus filiformis* with the assistance of transcriptome mining. Two characteristic peptides with a low homology in relation to known AMPs—the α-helical amphiphilic linear peptide, consisting of 28 amino acid residues and designated as HfBRI-28, and the 25-mer β-hairpin peptide, specified as HfBRI-25 and having a unique structure stabilized by two disulfide bonds—were obtained and analyzed as potential antimicrobials. Interestingly, both peptides showed the ability to kill bacteria via membrane damage, but mechanisms of their action and spectra of their activity differed significantly. Being non-cytotoxic towards mammalian cells and stable to proteolysis in the blood serum, HfBRI-25 was selected for further *in vivo* studies in a lethal murine model of the *Escherichia coli* infection, where the peptide contributed to the 100% survival rate in animals. A high activity against uropathogenic strains of *E. coli* (UPEC) as well as a strong ability to kill bacteria within biofilms allow us to consider the novel peptide HfBRI-25 as a promising candidate for the clinical therapy of urinary tract infections (UTI) associated with UPEC.

## 1. Introduction

Antimicrobial peptides (AMPs) are natural antibiotics produced by all living organisms, from archaea to mammals [1]. In multicellular organisms, they function as key factors of the innate immune system and act as the first line of defense against exogenous microbes such as bacteria, fungi, and protozoa or viruses invading the host.

Polychaeta is still an underexplored class of invertebrates in the context of discovery of new AMPs. Previously, we have identified β-hairpin AMPs arenicin-1 and -2 from the lugworm *Arenicola marina* [2], which are synthesized as the *C*-terminal part of the precursor proteins containing the so-called BRICHOS domain. This domain, consisting of ~100 amino acid residues, has been found in various living organisms from ancient marine invertebrates to humans [3]. In the last few years, many BRICHOS-containing proteins have been identified due to headway in genome sequencing. However, functions of many proteins belonging to the BRICHOS superfamily remain obscure. At the same time, the BRICHOS domain has been linked to some human diseases such as familial dementia (Bri2 and Bri3), Alzheimer’s disease (Bri2), respiratory disease (proSP-C), amyloidosis (Bri2, proSP-C), and cancer/tumor suppression (chondromodulin-I, gastrokines 1 and 2) [3]. One common attribute of all these known BRICHOS containing proteins (with the exception of proSP-C) is the presence of a C-terminal region with a high β-sheet propensity. It is likely that the major biological role of the BRICHOS domain is to act as an anti-amyloid chaperone, which obviously is a critical function for the correct folding of aggregation-prone precursors of hydrophobic AMPs.

To date, BRICHOS-related AMPs have been identified in a number of different marine worms. Interestingly, a single structural family of such peptides have been described for each species: β-hairpin arenicins from *A. marina* [2,4], β-hairpin abarenicins from *Abarenicola pacifica* [5], β-hairpin alvinellacins from sister species *Alvinella pompejana* [6] and *A. caudata* [7], β-hairpin capitellacin from *Capitella teleta* [8], α-helical nicomicins bearing a *C*-terminal loop stabilized by a disulfide bridge from *Nicomache minor* [9], α-helical (or dimeric β-sheet, exact structure is unknown) polaricin from *Amphitritides* sp. [10]. First described BRICHOS-related AMPs arenicins and alvinellacin have been directly isolated from the coelomocytes or coelomic liquid of animals [2,6]. Direct peptide isolation is a challenge due to the labor-consuming process of catching small solitary animals followed by the fine purification of peptides from immune cells and body fluids. Therefore, the *in silico* blasting of known sequences of various AMPs and their preprosequences in genome/transcriptome databases [5,6,8,10] or the RACE-based approach [9] were used to identify novel BRICHOS-related AMPs in further studies.

Our recent results [9] and the data obtained by Bruno et al. [10] have demonstrated that, in polychaetes, the BRICHOS domain might be involved in the biosynthesis of AMPs of different structural classes and not only of β-hairpin molecules. Interestingly, a detailed analysis of the *A. marina* transcriptome revealed two novel types of Cys-rich BRICHOS-related peptides designated as AmIMP1 and AmIMP2 (Integral Membrane Proteins) [11]. This fact provided an additional reassurance of the hypothesis of the BRICHOS domain function as a universal and auxiliary protein module facilitating biosynthesis of various relatively hydrophobic and cysteine-containing peptides, including host defense peptides. Moreover, the co-evolution of the BRICHOS domain structures and corresponding mature sequences seems to allow for the fine-tuning of the biosynthesis of AMPs under specific habitat conditions [7]. In this context, the BRICHOS-related AMP family from distributed worldwide and successfully adapted to extremely different microbe-laden environments marine annelids is not only an attractive model for the study of the host-pathogen co-evolution but is also a rich source of new AMPs. They are structurally shaped and adapted to function under specific conditions and exert their activities against diverse microbial pathogens. It can also be assumed that cosmopolitan animal species need a flexible defense system (including those based on BRICHOS-related AMPs) perfectly adapted to diversifying environmental pressures, such as microbial pathogen compositions and/or highly fluctuating abiotic factors (temperature, pH, salinity, and so on).

The marine polychaete *Heteromastus filiformis* (Claparède, 1864) has been found in various types of habitats around the world and referred to in many ecological studies [12]. This worm belongs to the phylum Annelida, the class Polychaeta, the subclass Sedentaria, the infraclass Scolecida, and the family Capitellidae. It has been found in Arctic, temperate, subtropical, and tropical seas. *H. filiformis* populations inhabit shallow estuaries and mudflats, and probably tolerate wide temperature and salinity ranges. *H. filiformis* was chosen as the object of this study. RNAseq data (NCBI accession no. SRX5588178) had previously been deposited for this animal, allowing the identification of all transcripts encoding the BRICHOS domain. As a result, a panel of all BRICHOS-containing translated sequences was identified. A detailed analysis of the obtained protein sequences with the use of phylogenetic analysis and 3D-modeling techniques revealed representatives of nine different families (one family consisting of three peptide isoforms, two families with each of them consisting of two peptides, and six families representing by a single peptide) of presumably host defense peptides with variable lengths from 18 to 91 amino acid residues. In this study, structures and biological activity of two selected novel BRICHOS-related AMPs from *H. filiformis* were systematically investigated.

## 2. Results and Discussion

### 2.1. The Marine Polychaete H. filiformis Is a Rich Natural Source of BRICHOS-Related Peptides

**Figure 1:**
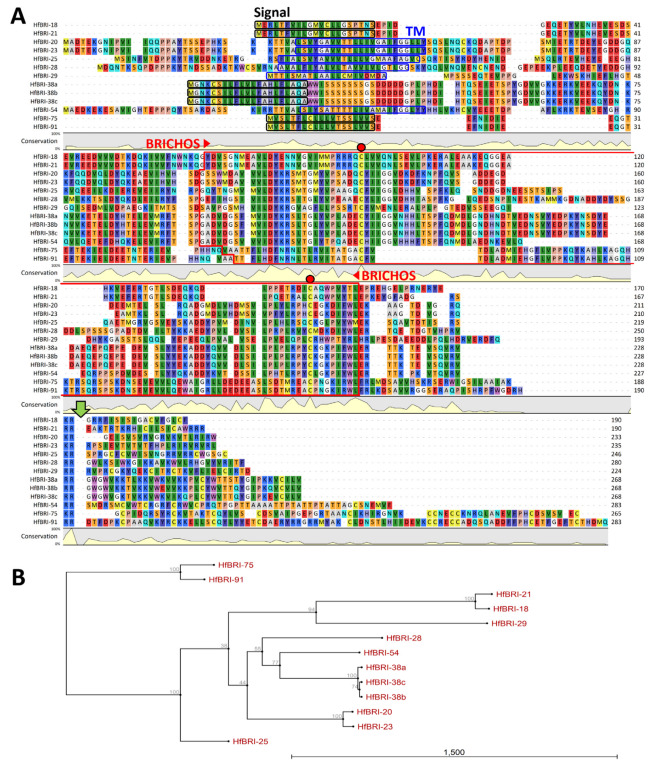
Amino acid sequence alignment (**A**) and a neighbor-joining phylogenetic tree (**B**) of the precursors of BRICHOS-related peptides from *H. filiformis*. The alignment and phylogenetic tree were constructed using CLC Sequence Viewer software (version 8.0). Bootstrap values are presented at the nodes. The values were obtained from 1000 replicates. Signal peptide sequences identified with SignalP 5.0 (https://services.healthtech.dtu.dk/service.php?SignalP-5.0), transmembrane parts (TM) identified with TMHMM-2.0 (https://services.healthtech.dtu.dk/service.php?TMHMM-2.0) and BRICHOS domain sequences identified with MyHits Motif Scan (https://myhits.isb-sib.ch/cgi-bin/motif_scan) are highlighted with black, blue, and red boxes, respectively. The access date: 13 June 2023. The conservative cysteine residues in the BRICHOS domain are marked with red circles. Putative post-translational processing dibasic sites are marked with a green arrow.

In this study, we performed a *de novo* assembly of *H. filiformis* transcriptome using the NCBI Sequence Read Archive (SRA, SRX5588178) data and identified 13 full-length transcripts encoding proteins that contain the BRICHOS domain followed by the dibasic motif Lys/Arg-Arg and a *C*-terminal AMP part (from 18 to 91 amino acid residues) enriched with positively charged residues (Figure 1). The dibasic motif preceding a putative propeptide cleavage site presupposed that a precursor might probably be activated by furin-like proteases, which is common for other BRICHOS-related AMP biosyntheses [9]. The discovered potential AMPs were named according to their origin and number of amino acid residues as HfBRI-18 (***H****eteromastus **f**iliformis* **BRI**CHOS-related **18**-residue peptide), -20, -21, -23, -25, -28, -29, -38a, -38b, -38c, -54, -75, and -91. Notably, the precursors of five peptides are transmembrane proteins consisting of three parts: a *N*-terminal short piece, a transmembrane helix, and a *C*-terminal part that includes the BRICHOS domain and a mature AMP sequence (HfBRI-20, -23, -25, -28, and -54). This is in sharp contrast to other HfBRI peptides which contain *N*-terminal signal sequences like most of known BRICHOS-related AMPs [9].

**Figure 2:**
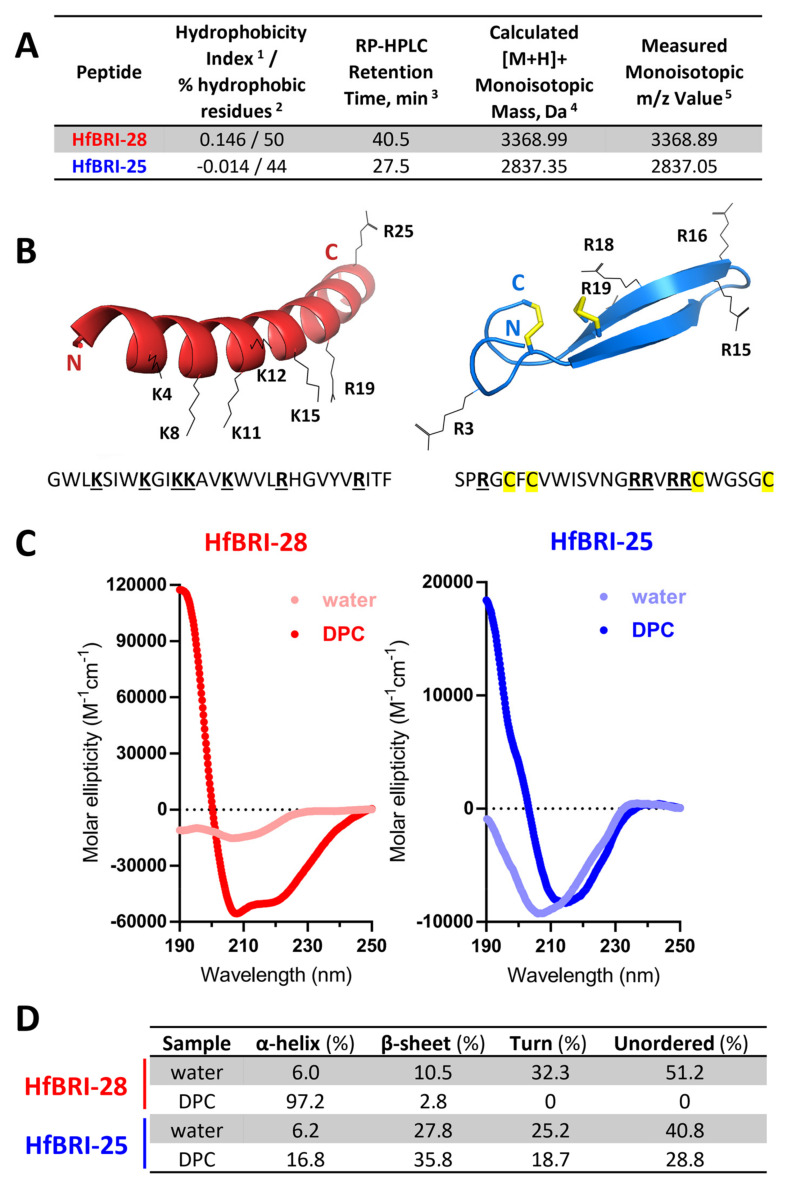
Structure analysis of the novel BRICHOS-related peptides HfBRI-25 and HfBRI-28. (**A**) Characteristics of the obtained peptides. ^1^ Mean Kyte–Doolittle hydrophobicity index (GRAVY) was calculated using the Expasy ProtParam tool. The maximum and minimum values of this index are +4.5 and −4.5 for poly-Ile and poly-Arg sequences, respectively. ^2^ Percentage of hydrophobic amino acids was calculated using the APD3 database. ^3^ Retention times were measured using semi-preparative reversed-phase high-performance liquid chromatography (RP-HPLC) on a C18 column with a linear gradient from 5 to 80% (*v*/*v*) acetonitrile in water containing 0.1% trifluoroacetic acid (TFA) within 50 min. ^4^ Molecular masses were calculated by considering the presence of four Cys residues forming two disulfide bonds in the case of HfBRI-25. ^5^ Molecular masses were determined experimentally using MALDI-TOF mass spectrometry. (**B**) Spatial structures of HfBRI-25 and HfBRI-28 were modeled using the AlphaFold algorithm (ColabFold server [20]). The top-rated models, according to predicted local distance difference test (pLDDT) scores, were visualized by the PyMOL software. (**C**) The CD-spectra of the peptides in water or in the presence of 30 mM DPC micelles. (**D**) The CONTINLL program [21] was used for CD data analysis.

The amino acid sequence alignment of prepropeptides (Figure 1A), phylogenetic analysis of these sequences (Figure 1B), as well as modeling the 3D structures of the predicted mature BRICHOS-related peptides (Figure S1 and Figure 2) allowed us to identify nine different structural families. Interestingly, all these identified BRICHOS-related peptides from *H. filiformis* are characterized by extremely low homology (less than 40–50%) with known AMPs according to search in the APD3 [13] and DRAMP [14] databases. We also attempted to search for BRICHOS-related AMPs by direct blasting of all *H. filiformis* ORFs against known peptide sequences from AMP databases: a total of 14,303 ORFs were predicted from the RNA-seq data, and 32 unique sequences were selected as candidate AMPs (Table S1) by using AMPml as well as online databases (CAMP and DBAASP). However, none of the BRICHOS-related peptides were detected using this approach. Thus, a methodology focused on the conserved domain-based bioinformatic search in genomes and transcriptomes may be a valuable alternative to AMP-based blasting to identify fundamentally new families of host defense peptides.

For the first time, two families of BRICHOS-related peptides without any cysteine residues were discovered. The smallest identified peptide, designated as HfBRI-18, contains the *C*-terminal 6-residue loop stabilized by one disulfide bond and known as the Rana-box motif which has been found in nicomicins from the polychaete *N. minor* [9] and earlier in a number of amphibian AMPs. Despite the presence of this structural similarity, the level of homology of HfBRI-18 with known AMPs did not exceed 40–50%. The Rana-box-like motif was also found in the central part of the peptide HfBRI-21; however, the formation of an intramolecular disulfide bridge is unfavorable according to the AlphaFold prediction (Figure S1), and the peptide is likely to form an amphipathic α-helix in a membrane environment. The third family consists of homologous peptides HfBRI-20 and HfBRI-23 (Figure 1), which probably form a highly amphiphilic β-hairpin structure stabilized by a salt bridge (Figure S1) instead of a single disulfide bond by analogy with arenicin-1 and -2 [2,4]. Interestingly, both antibacterial and cytotoxic activities have been shown to be similar in structure and length to artificial β-hairpin peptides consisting of the repeated (VR)_n_ motifs [15]. The linear peptide HfBRI-28 also does not contain cysteine residues but forms an amphiphilic α-helix projection (see Section 2.2). This peptide has a total positive charge of +7 at neutral pH, 50% hydrophobic residues, and resembles typical linear α-helical AMPs, despite its homology with the closest sequence analogs (amphibian AMPs of the caerin family) being less than 45%. The peptides HfBRI-25 and HfBRI-29, which belong to different HfBRI families, appear to form a β-hairpin structure and are stabilized by two disulfide bonds, presumably, with different Cys-pairing types.

The analysis of three different isoforms (a, b, and c) of the HfBRI-38 peptide showed the presence of two structural motifs, including the *N*-terminal α-helical region (residues 1–18) with a pronounced amphiphilicity, and the *C*-terminal β-hairpin region stabilized by a single disulfide bond. It is important to note that a high homology between the *N*-terminal regions of HfBRI-38 peptides and the 37-residue styelin-like AMP botryllin from the golden star tunicate *Botryllus schlosseri* was revealed [16]. The peptide HfBRI-54 also consists of two motifs: the *N*-terminal β-hairpin (residues 1–22) stabilized by two disulfide bonds, and the random coiled Thr-rich *C*-terminal region containing a single cysteine residue. It is interesting to note that the identical arrangement of cysteine residues in the *N*-terminal fragment (1–22) of HfBRI-54 and known AMPs tachyplesin and capitellacin (C-X_4_-C-X_4_-C-X_4_-C) has been revealed [8,17]. Thus, additional processing of the HfBRI-54 peptide cannot be excluded since its *N*-terminal fragment has all the characteristics of a typical β-hairpin AMP. Another discovered family includes the HfBRI-75 and HfBRI-91 peptides. Both of them contain 11 cysteine residues and do not possess a pronounced cationic property, a key characteristic of most AMPs. A low level of homology (about 30%) with known AMPs, a low accuracy of the predicted structures (predicted local distance difference test (pLDDT) value of 30–60) by the AlphaFold algorithm, as well as the absence of known domains and motifs in their structures (according to the SMART database [18]) do not allow us to make any reasonable assumptions regarding the structures or functions of these compounds.

### 2.2. Production and Structural Analysis of Novel Peptides HfBRI-25 and HfBRI-28

Among the 13 new found compounds, we selected 2 relatively small peptides for further studies: HfBRI-28, as a typical AMP-like molecule with a linear structure and pronounced cationic properties, as well as a presumably β-hairpin cationic peptide HfBRI-25 with a unique asymmetric arrangement of cysteine residues in its structure that distinguishes it from other known β-hairpin AMPs (Figure 2A,B). At the same time, similar to abarenicins, capitellacin, and alvinellacin, HfBRI-25 is stabilized by two disulfide bonds. We used bacterial expression systems for the heterologous production of HfBRI-25 fused with the modified thioredoxin A—a highly soluble carrier protein that was successfully used for the recombinant production of other β-hairpin AMPs stabilized by disulfide bonds [5,8,19]. The fusion protein was expressed in *E. coli* BL21 (DE3) or ClearColi BL21(DE3) cells, and the obtained total cell lysates were fractionated by affinity chromatography. After the purification and cleavage of the fusion protein, reverse-phase high-performance liquid chromatography (RP-HPLC) was employed to purify the target AMP. The peptide was then analyzed by MALDI-TOF mass spectrometry. The calculated [M + H]^+^ monoisotopic molecular mass corresponding to the HfBRI-25 amino acid sequence (2841.35 Da) exceeded the measured value (*m/z* 2837.05) by ~4 Da, indicating the formation of two disulfide bonds and the absence of any other modifications (Figure 2A and Figure S1).

The final yield of HfBRI-25 was of 4–6 mg/L of the culture for both expression strains, which was comparable to those of previously obtained β-hairpin AMPs [5]. The arrangement of disulfide bridges in HfBRI-25 structure was also elucidated by trypsin digestion followed by RP-HPLC and MALDI-TOF MS analysis (Figure S2). After digestion, we did not observe two terminal parts of HfBRI-25 that pointed at the absence of C1-C2/C3-C4 pairing type. Expectedly, the major product peaks appearing in the HPLC chromatogram (peaks 2 and 3, Figure S2) corresponded to two linear fragments (4–16) and (19/20–25) connected by two disulfide bonds. Therefore, the β-hairpin structure with the pairing type C1-C4/C2-C3 is the most probable, which agrees well with the AlphaFold prediction data (Figure 2B and Figure S3). Considering the simple linear structure of the peptide HfBRI-28 as well as the high hydrophobicity (due to a high ratio of aromatic residues) that may complicate its recombinant expression and purification, we applied solid-phase chemical synthesis followed by a two-step purification by RP-HPLC. As a result, HfBRI-25 and HfBRI-28 were obtained, and a purity level of >98% for both peptides was achieved.

The structures of HfBRI-25 and HfBRI-28 were studied by circular dichroism (CD) spectroscopy. To observe peptide structure changes upon interaction with lipid bilayers, we used zwitterionic dodecylphosphocholine (DPC) micelles as a membrane-mimicking environment. In contrast to isotropic mixtures of trifluoroethanol (TFE)/water, the detergent micelles have a lower propensity to distort spatial structures of solubilized proteins or peptides and do not induce the formation of artificial helical structures [22]. As shown in Figure 2C, the CD spectrum of HfBRI-28 dissolved in water showed a negative peak at ~205 nm, which indicated that the peptide mainly adopted random coil conformation. Therefore, this environment does not facilitate the peptide folding that is typical for linear AMPs. Despite the presence of five aromatic residues in the peptide sequence (which may contribute to the spectrum shape in the middle-UV region [23]), the CD spectroscopy of HfBRI-28 in the presence of DPC micelles showed a characteristic spectrum of α-helix with a strong positive peak at 190 nm and two negative peaks at 208 and 222 nm, which indicated that HfBRI-28 mainly adopted the α-helix conformation in membrane-mimicking environments. The secondary structure content analysis performed by the CONTINLL program also resulted in 97.2% of α-helix conformation (Figure 2D). According to the AlphaFold prediction data, five top-ranked models of the HfBRI-28 conformation (pLDDT average score > 70; Figure S3) corresponded to an amphipathic α-helix structure (Figure 2B). Peptides adopting the β-sheet conformation have a less intense and simpler characteristic CD spectrum, with a negative band at ~217 nm and a positive band at ~195 nm [23], while the negative CD band at ~210 nm is typical for β-turns [24]. Similar CD bands were observed for HfBRI-25 (Figure 2C). The spectrum of HfBRI-25 in water suggested the presence of a flexible chain, while an increase in the molar ellipticity of the band at ~190 nm and a small red shift from 205 nm to 215 nm in the presence of DPC indicated the formation of a more rigid and ordered structure in the membrane-mimicking environment. Notably, similar spectra in water and DPC were shown for protegrin-1, a well-known β-hairpin AMP from porcine leukocytes stabilized by two disulfide bonds [25].

### 2.3. HfBRI-25 Acts Selectively against Bacterial Cells

**Figure 3:**
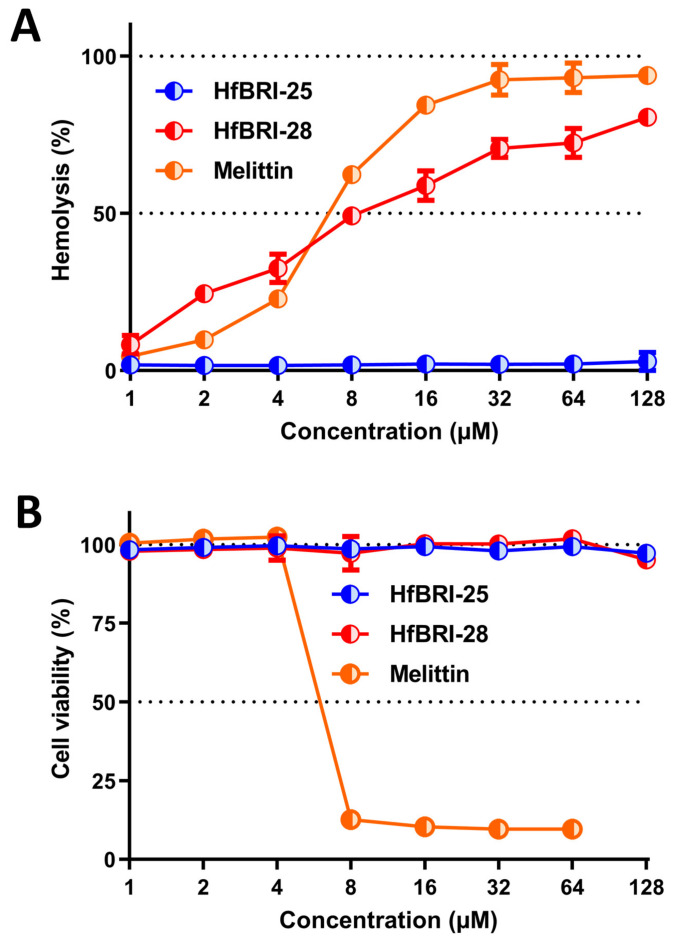
Biological activity of the peptides towards human cells. (**A**) Hemolytic effect on human red blood cells (hRBCs) after 2 h incubation (the hemoglobin release assay). (**B**) Cytotoxicity towards transformed human embryonic kidney cells (HEK293T) after 16 h incubation (the resazurin assay). The data are presented as the mean ± SD of two independent experiments.

The antibacterial activities of the obtained peptides were assessed by broth microdilution assay. The minimum inhibitory concentrations (MICs) were determined as the lowest concentrations of the peptides that prevented a visible growth of test strains of Gram-negative and Gram-positive bacteria in the Mueller–Hinton broth (MHB) (Table 1). The cytotoxic effects of these peptides were examined on human red blood cells (hRBCs) and transformed human embryonic kidney cells (HEK293T), as illustrated in Figure 3A,B, respectively. The spectrum of strains sensitive to HfBRI-25 in salt-containing media was limited to *E. coli* (MICs of 1 μM) and *Acinetobacter baumannii* (MICs of 1 μM). At the same time, the activity of HfBRI-25 was not inhibited by the presence of 5% fetal bovine serum (FBS) as compared with HfBRI-28. In particular, the MIC of HfBRI-25 towards *E. coli* ML-35p was of 1 μM in salt-containing MHB supplemented with 5% FBS, while the four- to eight-fold increase in MIC was observed for HfBRI-28 in these conditions. The same effect was also observed for HfBRI-25 in the presence of FBS at different concentrations up to 15% (MICs of 1 μM) that might point to a low tendency of the peptide to bind to serum proteins. Similar effects have been described earlier for several β-hairpin AMPs [26,27].

In contrast to HfBRI-25 and another α-helix AMP nicomicin from *N. minor* [9], the addition of 0.9% NaCl did not sufficiently alter the antibacterial activity of HfBRI-28 (Table 1). Moreover, we also found a strong specific activity of HfBRI-28 towards Gram-negative bacteria *Vibrio harveyi* (MIC of 0.06 μM), cultivating in the presence of 3% NaCl, mimicking marine salinity. *V. harveyi* is a well-recognized and serious bacterial pathogen of marine fish and invertebrates in aquaculture [28]. In other cases, this peptide exhibited an overall non-selective action against different bacteria, except for *S. aureus* ATCC 25923 and *Pseudomonas aeruginosa* ATCC 27853 strains where MICs exceeded acceptable values (>32 μM) and, thereby, were not estimated. Notably, both peptides showed considerable activities against fast-growing *Mycobacterium* sp., that is of particular interest with regard to more complex structure of the mycobacterial cell membrane together with previously defined low activities of cationic AMPs against these pathogens [29].

Being a highly amphipathic and hydrophobic peptide, HfBRI-28 produced a strong hemolytic effect on human red blood cells (hRBCs) comparable to that of the cytolytic agent melittin. Surprisingly, HfBRI-28 did not exhibit a cytotoxic activity towards mammalian cells at concentrations up to 128 μM. Such a difference may be due to both its strong interaction with the negatively charged surface of RBCs which contain sialic acid and interactions of the peptide with serum proteins added to the medium. In our previous study, a similar suppression of the cytotoxicity of another marine antimicrobial peptide UuBRI-21 in the presence of FBS was revealed [5]. Together, the above-mentioned properties of HfBRI-28 limited its possible application as a therapeutic agent, and in prospect, a significant structural optimization is required. In contrast, as a non-toxic at concentrations up to 128 μM, the peptide HfBRI-25 provides a significant therapeutic window and allows us to consider it as a selective candidate antibiotic.

### 2.4. HfBRI-25 and HfBRI-28 Kill Bacterial Cells via Membrane Damage

**Figure 4:**
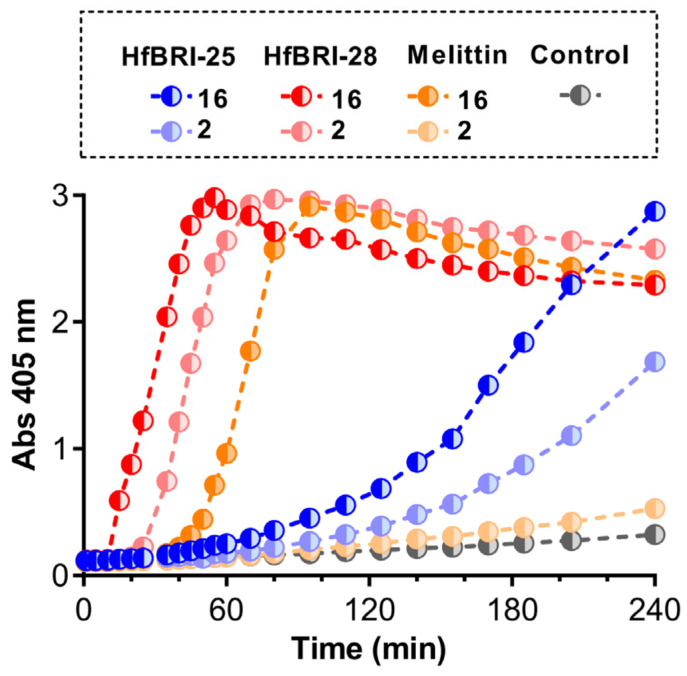
Kinetics of changes in the *E. coli* ML35p cytoplasmic membrane permeability measured with the use of ONPG (OD_405_) hydrolysis caused by the examined peptides. Peptide concentrations (μM) are presented within the dashed box. Control measurements were carried out without antimicrobial agents.

Cationic AMPs perform their antibacterial function by damaging membrane integrity and/or specifically inhibiting intracellular processes. In this study, an effect of the obtained peptides on bacterial cytoplasmic membrane integrity was analyzed with the use of *E. coli* ML-35p, the strain constitutively expressing β-galactosidase and lacking the functional lactose permease which is necessary for the uptake of o-nitrophenyl-β-D-galactoside (ONPG). When β-galactosidase cleaves ONPG, β-D-galactose and o-nitrophenol are released. The latter is a chromogenic product with absorbance at 405 nm. Both HfBRI-28 and melittin effectively damaged membranes at concentrations ≥ MIC values (Figure 4). In particular, HfBRI-28 caused almost full membrane permeabilization in 60 min at MIC (2 μM). Thus, the rapid membranolytic mode of action seems to be predominant for the HfBRI-28 peptide. In contrast, HfBRI-25, even at a concentration of 8 × MIC, demonstrated only a partial effect of cell membrane permeabilization after the same period. However, the delayed membrane damage effect of this peptide was observed at a concentration around the MIC, which points to a fundamentally different membrane-targeting mechanism of action of HfBRI-25 as compared to HfBRI-28.

**Figure 5:**
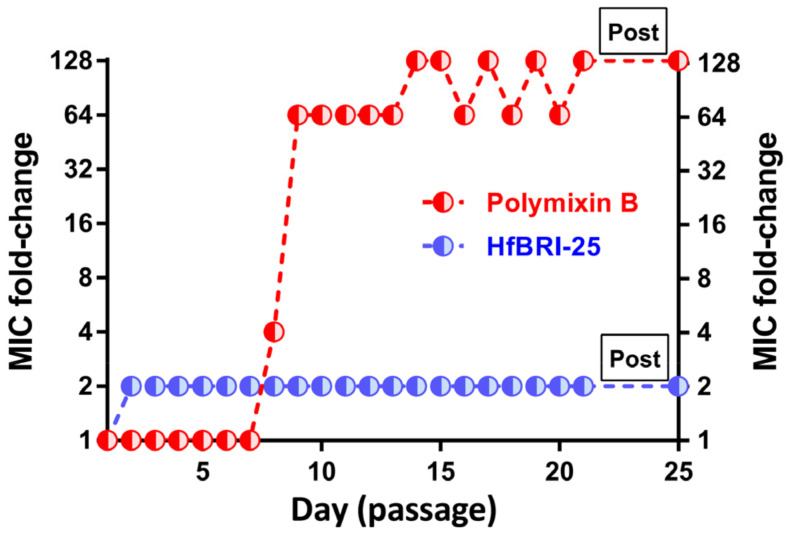
MIC changes in the bacterial strain *E. coli* MDR CI 1057 exposed to resistance selection by the peptide HfBRI-25 (initial MIC value = 1 μM) and by the reference antibiotic polymyxin B (PmxB, initial MIC value = 0.125 μM). The experiment was performed in the Mueller–Hinton broth supplemented with 0.9% NaCl at 37 °C. Bacteria that grew at the highest concentrations of AMPs after the final passage (on the 21st day) were further passaged three times (“Post”) on drug-free agar plates before determining the final MIC value. No differences in MICs before and after 21 passages without antimicrobial agents were observed.

The selection of resistant strains makes it possible to shed light both on key targets of the antibiotic and on mechanisms of protection against it in bacteria. In the next stage, the resistance of *E. coli* MDR Cl 1057 to HfBRI-25 was induced using day-to-day broth microdilution tests (Figure 5). This clinically isolated strain carries two well-characterized mutations in *gyrA* (S83L and D87N), causing high-level fluoroquinolone resistance as well as a higher-than-normal spontaneous mutation rate [19], and it has been used earlier in similar studies. As a result, the final MIC value differed only two-fold from the initial one even after 21 passages (days) in the medium supplemented with HfBRI-25. The following sub-culturing within three passages in the absence of the peptide showed that the final MIC was not changed.

Notably, polymyxin B rapidly induced a stable resistance and displayed a 128-fold increase in the MIC value, which matches well with our previous results [30]. It is believed that there is little or no development of resistance to cationic membrane-targeted AMPs without substantial fitness costs since this requires significant changes in structural and electrophysiological properties of the cell membrane. While the ability of HfBRI-25 to bind to some specific molecular targets on bacterial membranes or inside the cell is not excluded, it demonstrated a low tendency to induce a stable resistance in vitro that, together with membrane permeability data, pointed at a membranotropic mechanism of action. Being similar to another known β-hairpin AMP capitellacin, HfBRI-25 could demonstrate slow kinetics of membrane permeability [17]; however, the exact mechanism of interaction with bacterial membranes remains unknown and should be studied further.

### 2.5. In Vivo Efficiency and Stability of HfBRI-25 in Serum

**Figure 6:**
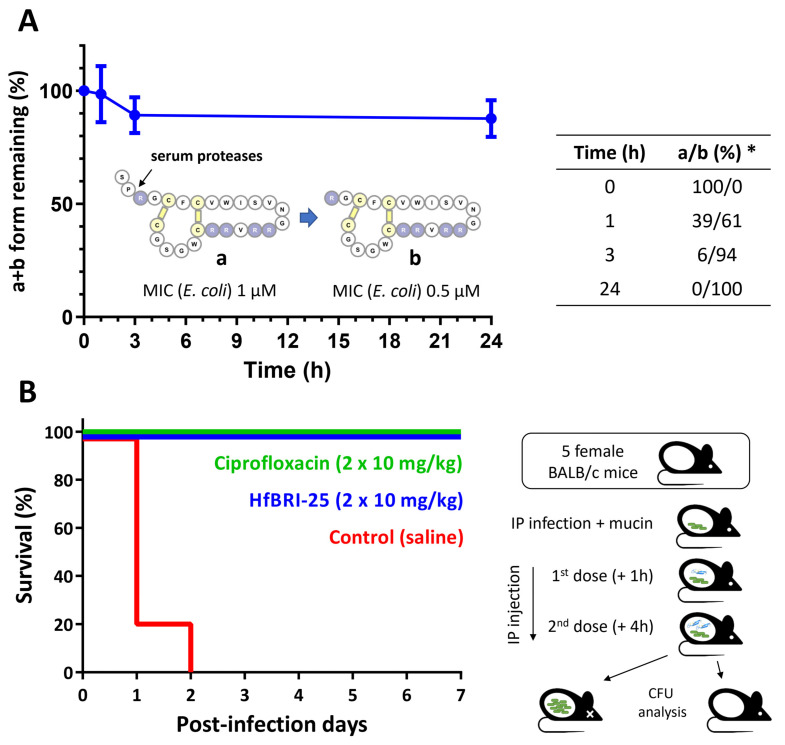
Stability in blood serum and *in vivo* efficiency of the peptide HfBRI-25. (**A**) Stability of HfBRI-25 in serum. The peptides were incubated at 37 °C in 25% buffered human serum and analyzed by RP-HPLC. * The ratio (a/b) of the peptide forms was estimated according to MALDI-TOF MS data. These data are presented as the mean ± SD of two independent experiments. (**B**) Survival rates of BALB/c mice (*n* = 5) infected intraperitoneally with *E. coli* ATCC 25922 (10^6^ bacteria in the presence of 2.5% mucin). Ciprofloxacin and saline were used as positive and negative controls, respectively. Health status of the examined mice was checked once a day for 7 days after infection.

The development of peptide-based therapeutics for systemic application is often limited by their rapid proteolytic degradation in the human circulatory system. Therefore, a high resistance of a peptide to proteolysis along with a low cytotoxicity are key properties of candidate compounds, indicating a possibility of entering the stage of preclinical in vivo studies by animal testing. Here, a degradation rate of HfBRI-25 was analyzed in 25% fresh human serum. To examine the cleavage patterns of the peptide in serum, the major fractions obtained by RP-HPLC after 0, 1, 3, and 24 h of incubation at 37 °C were analyzed by MALDI-TOF MS (Figure S5). The main product of HfBRI-25 degradation was the peptide without the *N*-terminal Ser^1^-Pro^2^ fragment located outside the Cys-stabilized β-hairpin part (Figure 6A). More than half of HfBRI-25 (form “a”) was converted into this 23-residue form “b” after 1 h of incubation in serum. This truncated form was additionally purified by RP-HPLC after 24 h of incubation with the serum, and its preliminary antibacterial activity screening against *E. coli* was performed. Surprisingly, the form “b” was shown to possess a slightly increased antimicrobial activity against the *E. coli* ML-35p or ATCC 25922 strains (MIC of 0.5 μM compared to MIC of the wild-type peptide of 1 μM). A similar two-fold increase in activity was shown for the capitellacin analog CT4 that lacked the *N*-terminal Ser-Pro fragment [17]. Notably, more than 80% of two active forms (a/b) remained intact in serum after 24 h (Figure 6A). These data demonstrated that two disulfide bridges provided the rigidity of the HfBRI-25 structure and its excellent stability in blood serum in vitro. Previously, the elevated rigidity of β-hairpin AMPs was shown to be important for their resistance to serum endoproteases [5,27].

An appropriate balance of the discovered advantages of HfBRI-25 (a low cytotoxicity, a high resistance to proteolysis, no serum influence on activity, a high antibacterial selectivity against *E. coli* bacteria) suggests that HfBRI-25 could be considered as a lead candidate suitable for in vivo testing as a systemic antibiotic against *E. coli*. Here, we examined the efficacy of HfBRI-25 in the mouse lethal septicemia model using five animals in a group (Figure 6B). To estimate animal survival rate, we applied the peptide two times (10 mg/kg in each injection) in the first day after infection. The intraperitoneal infection of BALB/c mice with *E. coli* ATCC 25922 in the presence of mucin resulted in the death of all mice within two days if treated using the vehicle control (saline) and the survival of all mice when treated with the control antibiotic ciprofloxacin. Here, we demonstrated a therapeutic efficacy of 100% for HfBRI-25 after a one-week experiment, which is similar to the results obtained earlier for another β-hairpin abarenicin analog, Ap9 [5]. A high CFU burden in mice from the negative control group was verified. Expectedly, we did not identify *E. coli* in spleen after euthanizing all surviving animals. These data suggested that natural unmodified animal β-hairpin AMPs can be comparatively safe and effective antimicrobials.

### 2.6. HfBRI-25 Effectively Kills Uropathogenic E. coli Strains and Embedded Cells inside Bacterial Biofilms

The ability of bacteria to form biofilms complicates the therapy of infectious diseases due to the resistance to antibiotic drugs. It is noteworthy that biofilms can be localized in host-organism tissues and colonize abiotic objects, such as surfaces of medical instruments. Urinary tract infections (UTIs) remain the most common biofilm-associated infections diagnosed in hospitalized patients. Notably, *E. coli* is the most widespread pathogen causing both community- and hospital-acquired UTIs [31]. Considering a high selectivity of the novel peptide HfBRI-25 against *E. coli* as well as its *in vivo* efficiency, its antibacterial activity was checked against a panel of clinically isolated uropathogenic *E. coli* (UPEC) strains, including MDR and XDR ones (Table 2 and Table 3). The peptide showed similar activities against all tested strains (MIC value of 1–2 μM) regardless of their resistance profile, an ability to produce biofilms, and the presence of serum in the medium. Moreover, the absence of cross-resistance was shown for HfBRI-25 when testing in vitro against selected PmxB-resistant *E. coli* MDR CI 1057 (Table 2, Figure 5). Interestingly, there were two strains with the MCR-1-mediated colistin resistance among bacteria sensitive to HfBRI-25 [32]. MCR-1 is a member of the phosphoethanolamine transferase enzyme family expressed in *E. coli* and responsible for modification of lipid A with phosphoethanolamine [33]. In particular, the MIC for polymyxin B increased 16-fold (from 0.125 to 2 μM) when acting on these strains. It seems that the mechanism of action of the peptide on *E. coli* membranes does not involve specific interactions with lipid A structure.

**Figure 7:**
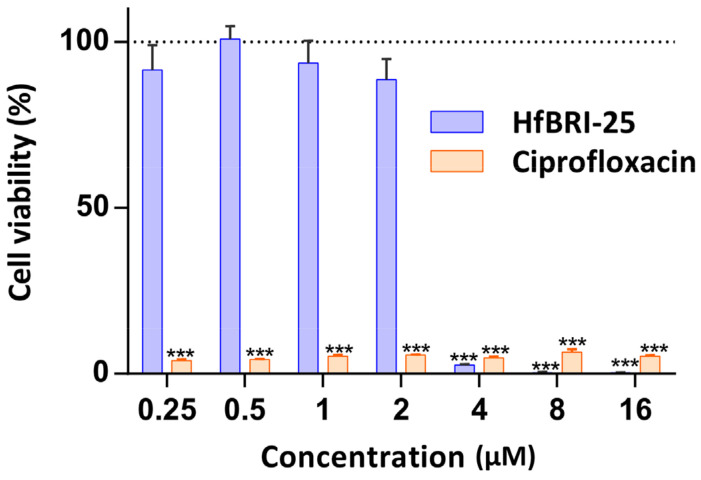
Impact of HfBRI-25 and conventional antibiotic ciprofloxacin on bacterial cell viability inside the biofilm formed by *E. coli* K12 SBS 1936. *** *p* < 0.001 (significantly different compared to the control).

Bacterial biofilms play an important role in UTIs, being responsible for their persistence. Therefore, the eradication of mature biofilms is an extremely challenging task because high concentrations or doses of antibacterial agents are required. Here, the ability of HfBRI-25 to disrupt established biofilms was evaluated as a number of viable bacteria in biofilm using the MTT assay. According to our previous data, *E. coli* K12 SBS 1936 was chosen as a strong biofilm-producer that can form it in different media [17]. The peptide showed a high activity against *E. coli* K12 SBS1936 biofilms even at 2 × MIC (4 μM), providing minimal residual bacterial viability (Figure 7). Ciprofloxacin, known as a strong anti-biofilm agent against corresponding susceptible bacterial strains [34], was used as a control and showed a high efficiency at a wide concentration range.

Our further efforts will be aimed at the study of the therapeutic efficacy of the peptide HfBRI-25 in UTI murine models, including those infected by multidrug-resistant UPEC strains. The found that a selectivity of action against *E. coli* strains might also be considered as an advantage of the HfBRI-25 peptide; however, potential negative effects against the human microbiome should be carefully analyzed.

## 3. Materials and Methods

### 3.1. Transcriptome Assembly and Prepropeptides Identification

The raw RNAseq data for *Heteromastus filiformis* was obtained from NCBI Sequence Read Archive (SRA). These data (accession number: SRX5588178) were converted to fastq format with a fasq-dump utility of sra-toolkit, and then the quality of reads was assessed using fastqc [35]. The raw reads for each sample were filtered to remove low-quality reads and adapter sequences using Trimmomatic software (v.0.38; [36]) with following parameters: ILLUMINACLIP:2:30:10, LEADING:30, TRAILING:30, SLIDINGWINDOW:10:25. The trimmed reads were then filtered from ribosomal RNA by aligning them to the SILVA database (release 138.1) using bowtie2 (v.2.3.5.1; [37]). Unaligned reads were used to assemble transcriptome with Trinity pipeline (v.2.9.1) with a minimum contig length of 200 bp. Assembled transcriptome was then directly translated and converted into protein blast database. The target prepropeptides were identified by blasting conservative regions of known BRICHOS domain sequences [8,9,10] against obtained database with BLASTP algorithm and also by searching for all translated sequences that contain BRICHOS domain in obtained database using HMMscan program [38].

### 3.2. Recombinant Production of HfBRI-25

The recombinant plasmid for expression of HfBRI-25 was constructed with the use of pET-based vector, as described previously [39]. The nucleotide sequences were designed on the basis of *E. coli* codon usage bias. The expression cassette was composed of the T7 promoter, the ribosome binding site, and the sequence encoding the chimeric protein that included His-tag, the *E. coli* thioredoxin A [M37L] (TrxL), methionine residue, and a mature peptide. *E. coli* BL21(DE3) cells were transformed with the corresponding plasmid and grown in lysogeny broth (LB) containing 20 mM glucose, 1 mM MgSO_4_, 50 mM Na_2_HPO_4_, 50 mM KH_2_PO_4_, 25 mM (NH_4_)_2_SO_4_, 100 μg/mL of ampicillin up to OD_600_ 0.7 ÷ 1 and then were induced with isopropyl β-D-1-thiogalactopyranoside (IPTG) at a final concentration of 0.2 mM for 16 h at 30 °C. The cultured cells were harvested by centrifugation and sonicated in the 100 mM phosphate buffer (pH 7.8) containing 20 mM imidazole and 6 M guanidine hydrochloride. The clarified lysate was loaded on a column packed with Ni-NTA agarose (GE Healthcare). The recombinant protein was eluted with a buffer containing 0.5 M imidazole. The eluate was acidified by concentrated hydrochloric acid, and the fusion protein was cleaved by excess of CNBr over methionine at 25 °C for 18 h in the dark. The lyophilized products of the cleavage reaction were dissolved in water and loaded on a semi-preparative Reprosil-pur C18-AQ column (10 × 250 mm, 5-μm particle size, Dr. Maisch GmbH). Reversed-phase high-performance liquid chromatography (RP-HPLC) was performed with a linear gradient of acetonitrile in water containing 0.1% TFA. The peaks were monitored at 214 and 280 nm, collected, and analyzed by MALDI-TOF MS using Reflex III mass-spectrometer (Bruker Daltonics). The fractions with corresponding molecular masses were dried in vacuo and dissolved in water. For animal experiments, the recombinant HfBRI-25 was produced in endotoxin-free ClearColi BL21(DE3) expression system with an additional repurification by RP-HPLC on a semi-preparative Reprosil-pur C18-AQ column. The trifluoroacetate (TFA) counterions were replaced with chloride ions by an incubation of the peptide with 5 mM HCl followed by lyophilization according to [40]. The peptide concentration was determined using near-UV absorbance at 280 nm and calculated extinction coefficients.

### 3.3. Chemical Synthesis of HfBRI-28

HfBRI-28 was synthesized by solid-phase peptide synthesis method on a scale of 0.1 mmol. A total of 250 mg of 2-chlorotrityl chloride resin (100–200 mesh, Iris Biotech, Marktredwitz, Germany) was swelled in dichloromethane (DCM) for 30 min in a polypropylene vessel [41]. The first amino acid was directly coupled to the resin using 2% N-methylmorpholine (NMM) in DCM, shaking for 1 h on an orbital shaker. The resin was then capped by adding methanol directly into the reaction vessel. Subsequent amino acids were coupled (without preactivation) using 0.4 mmol of Fmoc-protected amino acid, 0.4 mmol of HBTU coupling agent, 0.4 mmol of Oxyma Pure additive and 0.8 mmol NMM in dimethylformamide (DMF), shaking for 1 h [42,43,44]. Between amino acid couplings, the Fmoc-protecting group was removed via two 10 min agitations with 20% 4-methylpiperidine in DMF [45]. Removal of the final Fmoc-protecting group completed the peptide synthesis. Peptide was cleaved from the resin via a 2 h reaction with a cleavage cocktail consisting of 95:2.5:2.5 TFA/TIS/water [46]. Following cleavage, the resin was washed with TFA and DCM, and the volume of the cleavage solution was reduced by evaporation with nitrogen. The peptide solution was then transferred into cold methyl tert-butyl ether (MTBE) to precipitate the peptide. Peptide was pelleted by centrifugation, dried under vacuum, dissolved in 5% acetic acid, and lyophilized. Preparative RP-HPLC was performed with a linear gradient of acetonitrile in water containing 0.1% TFA on a Symmetry C18 column (19 × 300 mm, 5 μm, Waters, Eschborn, Germany) with a flow rate of 8 mL/min. Fractions with over 95% HPLC homogeneity were combined and lyophilized. The fraction with confirmed molecular mass by a MALDI-TOF MS was repurified by RP-HPLC on a semi-preparative Reprosil-pur C18-AQ column. Melittin (>98% pure) synthesized earlier using a standard solid-phase method was provided by Dr. Sergei Sychev. The peptide concentrations were determined using near-UV absorbance at 280 nm and calculated extinction coefficients.

### 3.4. Analytical Reversed-Phase HPLC

The fractions with confirmed masses were dried in vacuo and repurified using analytical reversed-phase HPLC to compare their retention times. Purification was performed using a Luna C18 column (4.6 × 150 mm, 5 μm particle size, Phenomenex) at a flow rate of 1 mL/min in a linear gradient of solution B2 (80% acetonitrile, 0.1% TFA) in solution A2 (5% acetonitrile, 0.1% TFA): 0–100% for 60 min.

### 3.5. Identification of Disulfide Pairing

The HfBRI-25 (0.1 mg) was dissolved in 0.1 mL of 100 mM Tris/HCl buffer (pH 8.0). Then, trypsin was dissolved in 50 mM acetic acid and added to the sample with enzyme-to-peptide ratio of 1:50 (*w*/*w*), followed by incubation at 37 °C for 16 h. The reaction was stopped by freezing. The tryptic digest of HfBRI-25 was loaded on an analytical Luna C18 column. RP-HPLC was performed with a linear gradient of acetonitrile (ranging from 0% to 40% for 40 min at a flow rate of 1 mL/min) in water containing 0.1% TFA. The major eluate fractions were lyophilized, dissolved in 0.1% TFA, and analyzed by MALDI-TOF MS.

### 3.6. Circular Dichroism Spectroscopy

Circular dichroism (CD) spectroscopy measurements were performed for peptides HfBRI-25 and HfBRI-28 dissolved in water or in dodecylphosphocholine (DPC) micelles. The final peptide concentration was 300 μM. The concentrated aqueous solution of DPC (Avanti Polar Lipids, Alabaster, AL, USA) was added to the peptide sample dissolved in water at a final peptide/lipid ratio of 1:100. Far-UV CD spectra were measured using a J-810 spectropolarimeter (Jasco, Tokyo, Japan) at 25 °C in demountable cells (Hellma, Mulheim/Baden, Germany) with 100 μm path length. Four consecutive scans were performed and averaged, followed by subtraction of the blank spectrum of the solvent.

### 3.7. Antimicrobial Assay

All the bacterial strains used in this study are listed in Table 3. The clinical isolates were collected and provided by Sechenov First Moscow State Medical University hospital and the State Collection of Pathogenic Microorganisms “Obolensk” (SRCAMB collection). The resistant to conventional antibiotics strains were defined as extensively drug resistant (XDR), according to [47]. Bacterial test cultures were grown in a Mueller–Hinton broth (MHB, Sigma) at 37 °C to mid-log phase and then diluted with the 2× MH medium supplemented with 1.8% NaCl (and, optionally, with fetal bovine serum (FBS, Gibco) at different concentrations) or without salt so that to reach a final cell concentration of 10^6^ CFU/mL. Moreover, 50 μL of the obtained bacterial suspension were added to aliquots of 50 μL of the peptide solutions serially diluted with sterilized 0.1% bovine serum albumin (BSA) in 96-well flat-bottom polystyrene microplates (Eppendorf #0030730011). After incubation for 24 h at 37 °C and 950 rpm on the plate thermoshaker (Biosan), minimum inhibitory concentrations (MIC) were determined as the lowest peptide concentrations that prevented growth of a test microorganism observed as visible turbidity. In most cases, no significant divergence of MIC values was observed (within ±1 dilution step). In the case of *Mycobacterium smegmatis*, MICs were determined in the Middlebrook 7H9 medium (HiMedia). The results were expressed as the median values determined on the basis of at least three independent experiments performed in triplicate.

### 3.8. Hemolysis and Cytotoxicity Assay

The hemolytic activity of the peptides was tested against the fresh suspension of human red blood cells (hRBC) using the hemoglobin release assay, as described previously [48]. Two experiments were performed with hRBC from blood samples of independent donors. The quantitative data are represented as average means with standard deviations. The cytotoxic effect of the peptides was tested on transformed human embryonic kidney cells (HEK293T) using the resazurin assay. The technique is based on the ability of live cell dehydrogenases to reduce a blue resazurin to pink and fluorescent resorufin. A total of 10^4^ cells per well in Dulbecco’s modified Eagle’s medium (DMEM/F12) supplemented with 10% FBS were placed into 96-well plates for 24 h in the CO_2_-incubator (5% CO_2_, 37 °C). Next, the culture liquid was replaced with the same fresh medium, in which the tested peptides were previously dissolved. After incubation for 16 h under these conditions, 20 μL of a solution of resazurin in sterile water (0.1 mg/mL) was added to each well, after which the incubation was continued for 4 h. Fluorescence of the formed resorufin was measured with the microplate reader AF2200 (Eppendorf, Germany) using the following channel: λ_Exc_ = 535 nm, λ_Em_ = 595 nm. The fluorescence signal in the wells containing cells cultured without the peptides was assumed to represent 100% cell viability. The experimental data were obtained from two independent experiments performed in triplicate.

### 3.9. Assessment of Bacterial Membrane Permeabilization

To examine the ability of the peptides to affect the barrier function of inner membrane of Gram-negative bacteria, we used the *E. coli* ML-35p strain constitutively expressing cytoplasmic β-galactosidase but lacking lactose permease. The state of the *E. coli* ML-35p cytoplasmic membrane was assessed based on its permeability to chromogenic marker o-nitrophenyl-β-D-galactopyranoside (ONPG, AppliChem), which is the β-galactosidase substrate. The cells were incubated in the trypticase soy broth (TSB) for 16 h at 37 °C, washed three times with phosphate-buffered saline (PBS, pH 7.4) to remove residual growth media, adjusted to the concentration of 2.5 × 10^8^ CFU/mL, and stored on ice until used. The assay was performed in PBS as well. The final concentration of *E. coli* ML-35p cells was 2.5 × 10^7^ CFU/mL. The final concentration of ONPG was 2.5 mM. Peptide samples were placed in the wells of a 96-well plate with a non-binding surface (Corning #3641), and optical density of the solution was enhanced due to the appearance of the hydrolyzed ONPG measured at 405 nm using a microplate reader AF2200. The final volume in each well was 200 μL. Assays were performed at 37 °C under stirring at 500 rpm. Control experiments were performed under the same conditions without adding a peptide. Two independent experiments were performed, and the curve pattern was the same.

### 3.10. Resistance Induction Assay

Resistance induction experiments were performed using the previously described method [20]. Briefly, on day one, the overnight culture of wild-type bacteria was diluted with the 2× MH broth supplemented with 1.8% NaCl to reach a final cell concentration of 10^6^ CFU/mL. Moreover, 50 μL of the obtained bacterial suspension were added to aliquots of 50 μL of the peptide solutions serially diluted with the sterilized 0.1% BSA in 96-well flat-bottom polystyrene microplates. After incubation for 20 ± 2 h at 37 °C and 950 rpm, MICs were determined, as described above. For each subsequent daily transfer, 2 μL of the inoculum taken from the first well containing a sub-inhibitory drug concentration were diluted with 2 mL of the fresh 2× MH broth supplemented with 1.8% NaCl. Then, 50 μL of this suspension were sub-cultured into the next passage’s wells containing 50 μL aliquots of the peptide at concentrations from 0.25× to 8–16× of the current MIC of each agent. A total of 21 repeated passages in the presence of antimicrobial agents were made for each bacterial strain during the experiment. Bacteria that grew at the highest concentration of AMPs on the final day were passaged an additional 3 times on drug-free agar plates before determining the final MIC value. Control serial passages in the absence of the agent were also included, and the resulting cultures showed unchanged MICs against antibacterial agents.

### 3.11. Stability in Serum

The serum stability of HfBRI-25 was determined in 25% human serum in PBS (pH 7.4) from healthy male donor, as described previously, with minor modifications [27]. Briefly, 20 μL of an aqueous peptide stock solution (5 mg/mL) was added to 480 μL of serum solution and incubated for 0, 1, 3, and 24 h at 37 °C. After incubation, serum proteins were selectively precipitated from the mixture by adding 10% TFA in the presence of 3 M urea. In the control samples (0 h), the precipitation was carried out immediately after the addition of serum. Subsequently, the samples were stored at 0 °C for 30 min and centrifuged at 30,000× *g* for 10 min. These samples were analyzed by analytical RP-HPLC (Symmetry 300 C18 column, Waters, Milford, MA, USA) and MALDI-TOF MS. Amount of the intact peptide was estimated from the corresponding peak area on the RP-HPLC chromatogram. Two independent experiments in duplicate were performed for each peptide.

### 3.12. The Effect of Peptides on Established Biofilm

*E. coli* K12 SBS1936 was grown in LB broth overnight at 37 °C and diluted in the same media to obtain 1 × 10^6^ CFU/mL. A total of 100 μL of bacterial suspension was added to each well of the 96-well polystyrene plate and grown at 32 °C and 120 rpm for 24 h. The wells were washed twice with sterile water to remove non-adherent cells. In parallel, 100 μL of peptide or ciprofloxacin solutions, serially diluted in LB medium using a separate 96-well polystyrene plate, were added to each well with pre-formed biofilm and incubated at 37 °C and 180 rpm for 16 h. Biofilm viability was assessed by using the MTT reagent (Sigma). After incubation, the wells were rinsed twice with sterile water and incubated with MTT solution (1 mg/mL in PBS) at 37 °C for 4 h. Next, the medium was removed. Formed formazan crystals were dissolved in a mixture of ethanol and dimethyl sulfoxide (1:1) for 10 min. Absorbance of each well was measured at 570 nm. Bacterial survival was calculated as a percentage relative to samples without antimicrobial agent. All experiments were conducted in at least two independent experiments in triplicate.

### 3.13. Animal Studies

Experiments were performed with female 8–10-week-old BALB/c mice (22–24 g; “Andreevka” laboratory animal nursery FMBA, Russia), according to the previously described method [27]. In brief, a total of 3 groups (5 mice per group) were infected by intraperitoneal (i.p.) injection of the bacterial inoculum (*E. coli* ATCC 25922), 10^6^ colony forming units (CFU) per animal, in the presence of 2.5% mucin (*w*/*v*). All these animals received two more i.p. injections at 1 h and 4 h after the bacterial challenge. The first group received ciprofloxacin (Sigma-Aldrich, St. Louis, MO, USA) as a positive control at a dose of 10 mg/kg per injection. The second group received the peptide HfBRI-25 at a dose of 10 mg/kg per injection. The third group received 0.9% NaCl (saline) as a vehicle control administered i.p. once (1 h post-infection). Survival was monitored for 7 days. After that, surviving animals were euthanized by CO_2_ asphyxiation. The spleen was aseptically removed, homogenized, serially diluted, and placed on Endo agar for CFU determination. All animal experiments were performed at the State Research Center for Applied Microbiology and Biotechnology (SRCAMB) in Obolensk (Russia). Experiments were approved by the Institutional Bioethics Committee of the SRCAMB (protocol number: 924/23, date of approval: 20 March 2023) and performed according to the Russian Federation’s rules and Directive 2010/63/EU of the European Parliament and of the Council.

## 4. Conclusions

According to the most recent data, the biosynthesis of a wide panel of different AMPs as mediated by universal and conserved precursor proteins is not a widespread phenomenon in nature. The most well-known example is the family of cathelicidins, whose precursors are produced in immune and epithelia cells and contain the *N*-terminal part of ~100 amino acid residues that is known as the cathelin-like domain (CLD) [49]. The CLD structure is highly conserved and is found in many vertebrates, whereas *C*-terminal parts, encoding mature peptides, show a substantial heterogeneity. Structures of mature cathelicidins include α-helices, β-hairpins, and extended linear regions enriched with Trp or Pro/Arg residues [30,50]. Apparently, a similar biosynthesis system and platform for the evolution of defense peptides may function in polychaetes producing BRICHOS-related AMPs. Presumably, the BRICHOS domain serves as an intramolecular chaperone, preventing the aggregation of hydrophobic regions and promoting the formation of correct spatial structures, including native conformations of hydrophobic AMPs, during biosynthesis. Here, nine new structural families of BRICHOS-related peptides with an extremely low homology with known AMPs were discovered in the cosmopolitan marine worm *H. filiformis*. As proof of the concept, we synthesized two of them and showed their high antimicrobial activity against a wide panel of Gram-negative and Gram-positive bacteria.

To date, RNAseq data have been deposited in the NCBI Sequence Read Archive (SRA) for >300 polychaete species, which opened the way to the discovery of dozens or even hundreds of new families of BRICHOS-related AMPs, primarily those stabilized by disulfide bonds. Obviously, only a minor part of them (due to their high hydrophobicity and tendency toward aggregation [9]) will possess the desired set of properties to become antimicrobial drug candidates. Meanwhile, this approach may significantly expand the pool of protease-resistant molecular scaffolds in the near future. Here, we found that the natural β-hairpin HfBRI-25 peptide without any sequence optimization or modification by medicinal chemistry methods had all the necessary properties of a selective antibiotic agent, such as (**i**) a high activity against a wide panel of *E. coli* strains in the presence of serum, including those in biofilm matrix; (**ii**) the absence of hemolytic and cytotoxic properties; (**iii**) a high resistance to serum proteases; and (**iv**) a high efficacy *in vivo*. Moreover, the truncated 23-residue analog can be considered as an optimized form of HfBRI-25 with an increased activity. In total, this allows us to consider HfBRI-25 and its analog as lead candidate peptides for the development of a last-resort systemic antibiotic for the treatment of multidrug-resistant UPECs in patients with a poor response to standard antibiotic therapy against UTI with known etiology. HfBRI-25 remained effective against colistin-resistant strains and might be a promising agent for the treatment of clinically and genotypically diverse *E. coli* strains, including infections associated with colistin-resistant bacteria. Our future efforts will focus on expanding *in vivo* efficacy tests of this peptide, using animal models of infections caused by UPECs resistant to all known antibiotics, including polymyxins. In addition, the unique β-hairpin structure of HfBRI-25 with asymmetric disulfide bonds may be of interest to those in the field of peptide engineering and “molecular grafting” [51] as a proteolysis-resistant scaffold for the development of various peptide therapeutics.

## Figures and Tables

**Table 1:** Antibacterial activities of the obtained peptides.

Bacteria	Minimum Inhibitory Concentration (μM)			
	HfBRI-25		HfBRI-28	
	MHB	MHB + 0.9% NaCl	MHB	MHB + 0.9% NaCl
**Gram-negative**				
*Escherichia coli* ML-35p	0.25	1	2	2
*Escherichia coli* ATCC 25922	0.5	1	2	1
*Acinetobacter baumannii* XDR CI 2675	0.125	1	2	2
*Enterobacter cloacae* XDR CI 4172	1	16	0.5	2
*Klebsiella pneumonia* ATCC 700603	2	32	16	16
*Pseudomonas aeruginosa* ATCC 27853	2	>32	32	>32
*Vibrio harveyi* BB120	1 *		0.06 *	
**Gram-positive**				
*Staphylococcus aureus* ATCC 6538P	2	>16	8	8
*Staphylococcus aureus* ATCC 29213	n.d.	>16	n.d.	>32
*Bacillus mycoides* VKM B-814	0.5	32	0.5	2
*Bacillus subtilis* VKM B-886	1	4	1	0.5
*Bacillus licheniformis* VKM B-511	1	16	8	4
*Mycobacterium phlei* Ac-1291	2	16	2	1
*Mycobacterium smegmatis* mc(2)155	1 **		1 **	

* MICs were determined in LB medium supplemented with 3% NaCl; ** MICs were determined in the 7H9 Middlebrook broth.

**Table 2:** Antibacterial activity of HfBRI-25 against *E. coli* strains.

Bacteria	Minimum Inhibitory Concentration (μM)		
	MHB	MHB + 0.9% NaCl	MHB + 0.9% NaCl + 5% Serum
*E. coli* ML-35p	0.25	1	1
*E. coli* ATCC 25922	0.5	1	2
*E. coli* ATCC 23724	0.5	1	2
*E. coli* K12 BW25113	0.125	1	2
*E. coli* K12 SBS 1936	0.5	2	2
*E. coli* CI 214	0.25	1	1
*E. coli* XDR CI 3600	0.5	2	2
*E. coli* MDR 1057	0.5	1	1
*E. coli* MDR 1057 (PmxB-res) *	1	2	2
*E. coli* XDR U10 (*mcr-1^+^*)	0.125	1	1
*E. coli* XDR U22 (*mcr-1^+^*)	0.125	1	1

* The strain was obtained in this study (see Figure 5).

**Table 3:** Bacterial strains used in this study.

Bacterial Strain	Characteristics (Source, Antibiotic Resistance)
*Escherichia coli* DH10B	Cloning strain (Invitrogen)
*Escherichia coli* BL21 (DE3)	Expression strain (Novagen)
*Escherichia coli* ClearColi BL21(DE3)	Expression strain (Research Corporation Technologies)
*Escherichia coli* ML-35p	Laboratory strain (ATCC collection)
*Escherichia coli* ATCC 25922	Laboratory strain (ATCC collection)
*Escherichia coli* ATCC 23724	Laboratory strain (ATCC collection)
*Escherichia coli* K12 BW25113	Laboratory strain (Keio collection)
*Escherichia coli* K12 SBS 1936	Laboratory strain (IMG RAS collection)
*Escherichia coli* U10 SRCAMB B-8551	XDR clinical isolate (urine, kidney stone disease; ESBL+; mcr-1+)
*Escherichia coli* U22 SRCAMB B-8553	XDR clinical isolate (urine, UTI; ESBL+; mcr-1+)
*Escherichia coli* 1057 SRCAMB B-10910	MDR clinical isolate (urine, UTI; ESBL+)
*Escherichia coli* (MDR CI 3600)	MDR clinical isolate (urine, UTI; ESBL+)
*Escherichia coli* (CI 214)	Clinical isolate (urine, acute pyelonephritis)
*Enterobacter cloacae* (XDR CI 4172)	XDR clinical isolate (MBL+)
*Acinetobacter baumanii* (XDR CI 2675)	XDR clinical isolate (MBL+)
*Klebsiella pneumoniae* ATCC 700603	Laboratory strain (ATCC collection)
*Pseudomonas aeruginosa* ATCC 27853	Laboratory strain (ATCC collection)
*Vibrio harveyi* BB120	Laboratory strain (ATCC collection)
*Bacillus subtilis* B-886	Laboratory strain (VKM collection)
*Bacillus licheniformis* VKM B511	Laboratory strain (VKM collection)
*Bacillus mycoides* VKM	Laboratory strain (VKM collection)
*Staphylococcus aureus* ATCC 29213	Laboratory strain (ATCC collection)
*Staphylococcus aureus* ATCC 6538P	Laboratory strain (ATCC collection)
*Mycobacterium smegmatis* mc(2)155	Laboratory strain (ATCC collection)
*Mycobacterium phlei* Ac-1291	Laboratory strain (VKM collection)

CI, clinical isolate; UTI, urinary tract infection; MDR, multidrug-resistant strain; XDR, extensively drug-resistant strain; ESBL+, extended spectrum beta-lactamase-producing strain; MBL+, metallo-beta-lactamase-producing strain; mcr-1+, *mcr-1* gene-mediated resistance to polymyxins (colistin and PmxB).

## Data Availability

Data are contained within the article and Supplementary Materials.

## Supplementary Materials

The following supporting information can be downloaded at: https://www.mdpi.com/article/10.3390/md21120639/s1, Figure S1: Structure analysis of those novel BRICHOS-related peptides from *H. filiformis*; Figure S2: MALDI-TOF MS analyses of the obtained peptides HfBRI-25 and HfBRI-28; Figure S3: Accuracy of the predicted structures (pLDDT) for both peptides HfBRI-25 and HfBRI-28; Figure S4: RP-HPLC and MALDI-TOF MS analyses of the tryptic digests of HfBRI-25; Figure S5: Stability of HfBRI-25 in serum (RP-HPLC and MS data); Table S1: A list of predicted non-BRICHOS-related AMPs from the *H. filiformis* RNA-seq data.
