# Supplementary material for: Novel BRICHOS-Related Antimicrobial Peptides from the Marine Worm Heteromastus filiformis: Transcriptome Mining, Synthesis, Biological Activities, and Therapeutic Potential
Source: Mar Drugs. 2023 Dec 14;21(12):639. doi: 10.3390/md21120639 (PMC10745061; doi:10.3390/md21120639)
Supplement: Supplementary file 1 [file marinedrugs-21-00639-s001.zip › marinedrugs-2754393-supplementary.pdf]

Supplementary Materials

Novel BRICHOS-related antimicrobial peptides from the marine worm *Heteromastus filiformis*: transcriptome mining, synthesis, biological activities, and therapeutic potential

Pavel V. Panteleev[1], Victoria N. Safronova[1], Shuting Duan[1,2], Alexey S. Komlev[3], Ilia A. Bolosov[1], Roman N. Kruglikov[1], Tatiana I. Kombarova[4], Olga V. Korobova[4], Eugenia S. Pereskokova[4], Alexander I. Borzilov[4], Igor A. Dyachenko[5], Olga V. Shamova[3], Yu Huang[2], Qiong Shi[2,6] and Tatiana V. Ovchinnikova[1,7*]

[1] M.M. Shemyakin & Yu.A. Ovchinnikov Institute of Bioorganic Chemistry, Russian Academy of Sciences, 117997 Moscow, Russia; p.v.panteleev@gmail.com (P.V.P.); victoria.saf@ibch.ru (V.N.S.); bolosov@ibch.ru (I.A.B.); kruglikov1911@mail.ru (R.N.K); ovch@ibch.ru (T.V.O.);

[2] Shenzhen Key Lab of Marine Genomics, Guangdong Provincial Key Lab of Molecular Breeding in Marine Economic Animals, BGI Academy of Marine Sciences, BGI Marine, 518081 Shenzhen, China; huangyu@genomics.cn (Y.H.), shiqiong@genomics.cn (Q.S.);

[3] Institute of Experimental Medicine, WCRC "Center for Personalized Medicine" 197022 St. Petersburg, Russia; komlev1420@yandex.ru (A.S.K.), oshamova@yandex.ru (O.V.S);

[4] State Research Center for Applied Microbiology & Biotechnology (SRCAMB), 142279 Obolensk, Russia; kombarova@obolensk.org (T.I.K.); korobova@obolensk.org (O.V.K.); pereskokova@obolensk.org (E.S.P.), borzilov@obolensk.org (A.I.B.);

[5] The Branch of M.M. Shemyakin & Yu.A. Ovchinnikov Institute of Bioorganic Chemistry, Russian Academy of Sciences, 142290 Pushchino, Russia; dyachenko@bibch.ru (I.A.D.);

[6] Laboratory of Aquatic Genomics, College of Life Sciences and Oceanography, Shenzhen University, 518057 Shenzhen, China; shiqiong@szu.edu.cn (Q.S.).

[7] Department of Biotechnology, I.M. Sechenov First Moscow State Medical University, 119991 Moscow, Russia

* Correspondence: ovch@ibch.ru

Peptide	Sequence	Predicted spatial structure	Predicted fold type
HfBRI-18	GRRFISISIGACVFGLCF	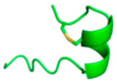	"Rana-box"-like (one disulfide bond)
HfBRI-21	EAKTRTKRHICILSICAWRRR	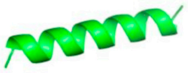	linear α-helical
HfBRI-20	**GEISVSVRVGRVKVTLRIRW**	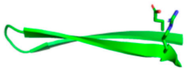	β-hairpin
HfBRI-23	**RPSIEVTVTVTFHPLRIRVRVRL**	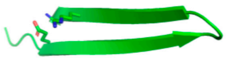	β-hairpin
HfBRI-29	RVPRCGKYQEKC**ITRCTKVFLIELCIRTD**	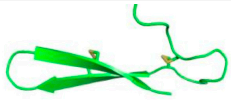	β-hairpin + loop (two disulfide bonds)
HfBRI-38a	GWGWVKKTLKKVWKVVKKP<br>**VCYWTTSTYGIPKKVCILV**	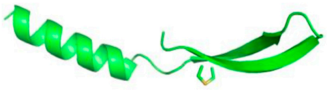	α-helix + β-hairpin (one disulfide bond)
HfBRI-54	SMD**RSMCVWTCRGRFCRWVCPR**<br>QTPGPTTAAAATTPTATTPTAT<br>TAGCSNEMVE	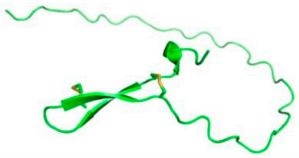	β-hairpin + random coiled Thr-rich part (two disulfide bonds)
HfBRI-75	GCPIDQRSYRCKVTAKTCQYLV<br>SCDSVAIPGEPGRTAANCIKHI<br>RGNVKCCNECCKNRQLANEVFP<br>HCDSVSVEC	n.d.	cysteine-rich
HfBRI-91	DTFDPKCPAAQVKYRCKKELLSCQY<br>LYYETCDAERYRRGRRMYAKCLDNS<br>TLHIIDEVKCCRECCADQSQADDFF<br>PHCETFGEFTCTHDMQ	n.d.	cysteine-rich

Figure S1. Structure analysis of those novel BRICHOS-related peptides from *H. filiformis*. Spatial structures of the peptides were modelled using the AlphaFold algorithm (ColabFold server https://colab.research.google.com/github/sokrypton/ColabFold/blob/main/AlphaFold2.ipynb). Accuracy of the predicted structures (predicted local distance difference test (pLDDT)) is not indicated. The top ranked models, according to pLDDT scores, were visualized by the PyMOL software and presented as predicted spatial structures. β-Hairpin motif sequences are marked with bold.

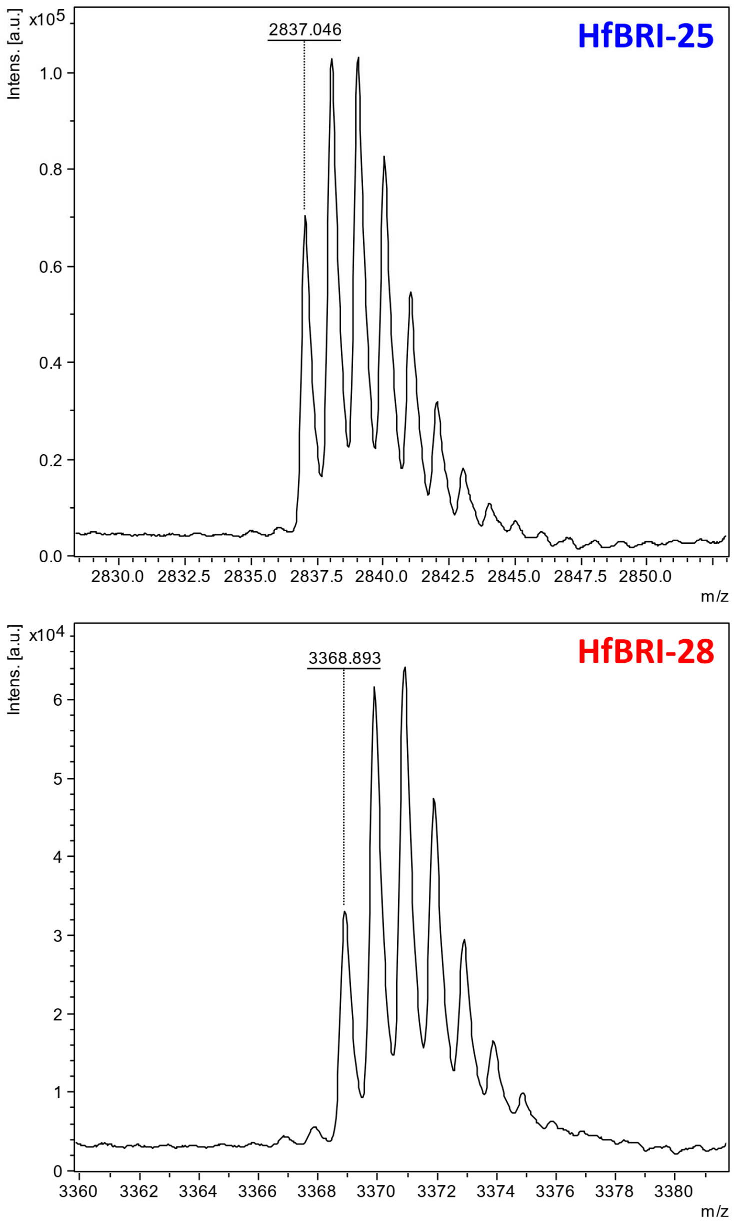

Figure S2. MALDI-TOF MS analyses of the obtained peptides HfBRI-25 and HfBRI-28.

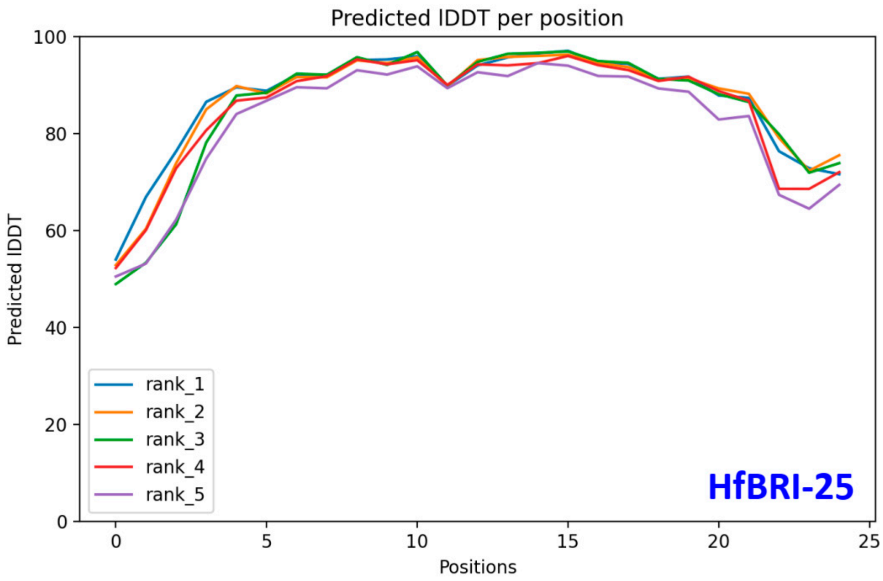

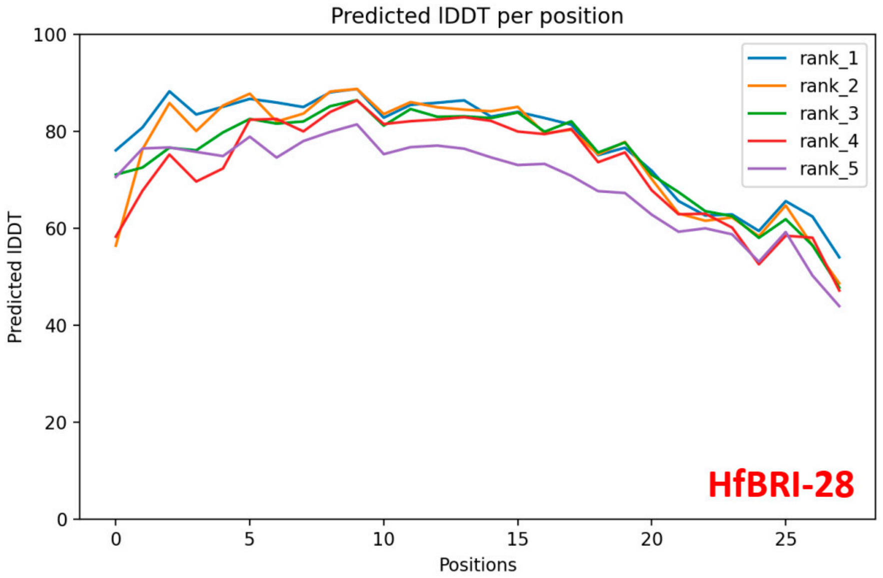

Figure S3. Accuracy of the predicted structures (pLDDT) for both peptides HfBRI-25 and HfBRI-28.

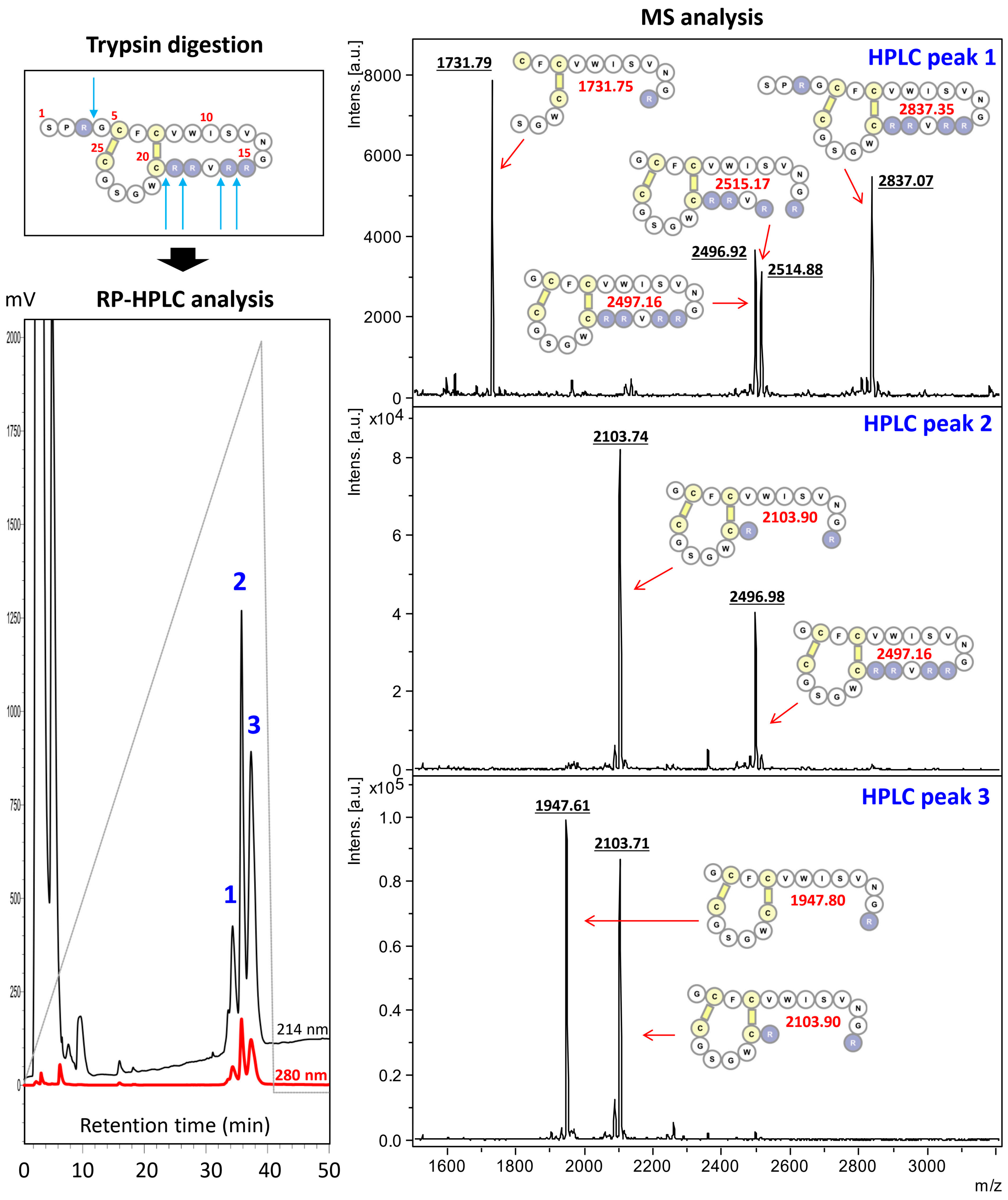

Figure S4. RP-HPLC and MALDI-TOF MS analyses of the tryptic digests of HfBRI-25. The experimentally measured monoisotopic $[M+H]^+$ values of the major RP-HPLC fractions pointed by the numbers (colored in blue) matched well the corresponding calculated values (colored in red).

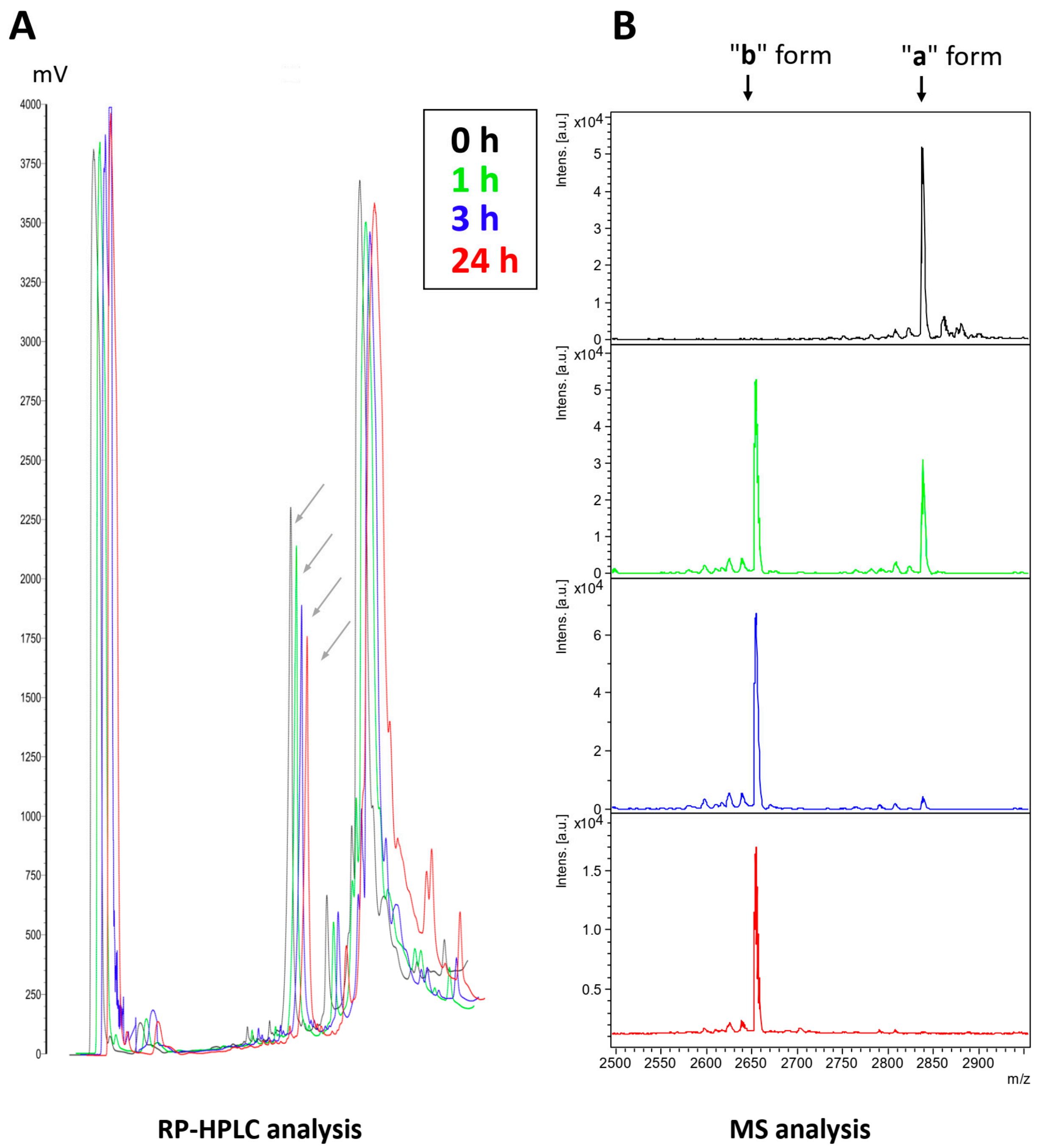

Figure S5. Stability of HfBRI-25 in serum (RP-HPLC and MS data). **(A)** RP-HPLC profiles (Abs 214 nm) of fractions containing HfBRI-25 and its shortened 23-residue form after 0, 1, 3, and 24 h incubation in 25% buffered human serum. Fractions containing mixed target peptides (HfBRI-25 ("a" form) and its shortened 23-residue "b" form) are marked with grey arrows. **(B)** MALDI-TOF MS analysis of the target RP-HPLC peaks containing HfBRI-25 and its shortened 23-residue form after 0, 1, 3, and 24 h incubation in 25% buffered human serum.

Table S1. A list of predicted non-BRICHOS-related AMPs from the *H. filiformis* RNA-seq data

Predicted AMP #	Sequence
1	LISGMRGLGVEIAKNVILAGVKSVTIHDTGN
2	GRVEKFIQKSERRIFTNFH
3	YVPKVVGLYTVTVTYGGKPIKKSPFKVEVSLAFDSSKVYATGRG
4	LDIWDKVHEALHTLIKLAKKGLTAKHS
5	LVTNPKRVQTGIDKKACNCLLLKVN
6	LWEIGKIIGQMWR
7	RALVTAVHSVPGVASLPAGSIKAALTVVQRYPQCLGYNWGP
8	RHIKIWFQNRRMKWKK
9	LSVKVAPTIFGTVTK
10	EAAPLARGASNLSKRASTITKKASTLTKKA
11	RGASNLSKRASTIAKKASTLTKKASLAKQA
12	LVMERKWMIRARHIAGK
13	FTKNCIVLFPGHPIVHGRENSARGFRNLTDLF
14	AVTPKVATKDSKKPS
15	GKHITGLQMGSNKGASQAGTGFGRPRQV
16	RKYRNAHKRKLQKKEYRNLQKIVPSVAEKS
17	EHGRRLERLAKGFFPSSEKACPAFLRHK
18	KLLRLQRKIAQQKRKKY
19	LSRNYRKRIERLKKLKKKRPAVRW
20	GLARGLHECAKALDK
21	VKLKRKAKALISRMKSNISK
22	LAGVTKSLQDLASRFAGL
23	MAKTILKRLFRVYAHIY
24	GSGPVRSSCLRGSSHSPSAVHHCISEAIHKQ
25	IRGKSQSVKSPVKGKLPSIRTSPVKGETSYVKNNP
26	FFQTSWNKLGNEDRQCLKGMKTKGARFGTAL
27	GFGSALFSIGRKLIPKILP
28	TTILQSVGKQFAKNIFGILQSIKAREEGS
29	RLLTRIVKFIWRFVRRL
30	GCRQALGRARRRLRNLGRW
31	IKVGINGFGRIGRLV
32	EDLKKIIQKIKSHYK
